# Influence of Carbon Nanotubes on Phase Composition, Thermal and Post-Heating Behavior of Cementitious Composites

**DOI:** 10.3390/molecules26040850

**Published:** 2021-02-06

**Authors:** Mohammad R. Irshidat, Nasser Al-Nuaimi, Mohamed Rabie

**Affiliations:** Center for Advanced Materials (CAM), Qatar University, P.O. Box 2713, Doha, Qatar; anasser@qu.edu.qa (N.A.-N.); mr1300307@student.qu.edu.qa (M.R.)

**Keywords:** carbon nanotubes, cement mortar, fire, thermal, strength, microstructure

## Abstract

This paper experimentally investigates the influence of carbon nanotubes (CNTs) on phase composition, microstructure deterioration, thermal behavior, and residual mechanical strengths of cementitious composites exposed to elevated temperatures. Cement mortars with small dosages of CNTs, 0.05% and 0.2% by weight of cement, were prepared and then heated at 25 °C, 150 °C, 200 °C, 450 °C, and 600 °C for two hours before being tested. The results show positive impact of the CNTs on the hydration process of cement mortar at room temperature and at higher temperatures up to 200 °C. Decomposition of the hydration products is obvious at 450 °C, whereas sever deterioration in the microstructure occurs at 600 °C. The nano reinforcement and bridging effect of the CNTs are obvious up to 450 °C. Thermal behavior characterization shows that CNTs incorporation enhances the thermal conductivity of the unheated and heat-treated mortar specimens. The decomposition of the hydration products needs more heat in the presence of CNTs. Finally, presence of CNTs significantly enhances the residual compressive and flexural strengths of heated mortar specimens for all studied temperatures.

## 1. Introduction

Concrete is the most used manmade materials in the construction industry all over the world. However, its low tensile strength represents the major drawback that causes many durability problems. To enhance the durability of cementitious composites, the researchers focused on improving the properties of cement matrix through using various additives such as silica fume and fly ash, or fiber reinforcements such as steel and polypropylene fibers [1,2]. Usually, the initiation of the cracks in concrete starts at nano level. Carbon nanotubes (CNTs) with their small dimensions and tremendous properties represent one of the most promising additives that can not only delay the initiation of the cracks, but also control their propagation. CNTs have excellent mechanical and thermal performance. The tensile strength, tensile failure strain, and elastic modulus of CNTs can be predicted to be almost 500 GPa, 30%, and 1 TPa, respectively [3,4]. CNTs’ thermal conductivity is almost nine times that of copper [5]. The potential of utilizing these excellent mechanical and thermal properties of CNTs to modify cementitious materials received significant attention in the last decade. Many studies showed that adding CNTs to the cementitious materials accelerated the hydration of cement [6,7]. They claimed that CNTs acted as nucleation for the hydration products, which accelerated their crystallization process and thus the overall hydration process. Other studies showed that incorporating CNTs in cementitious composites was an efficient way to improve their mechanical properties [8,9,10] such as compressive and flexural strengths [11,12,13,14,15], fracture toughness [16], and Young modulus [4]. CNTs improved these properties by bridging the nano-cracks and delaying their propagation to micro level [17]. Shi et al. [18] and Rashad [19] recently published review papers summarizing the up-to-date research conducting on utilizing CNTs in cementitious composites. They concluded that most of the studies focused on investigating the effect of CNTs on the mechanical properties of cement-based materials. The effect of such additives on the durability and the behavior of cementitious composites when subjected to aggressive conditions such as fire was rarely studied.

Exposure to fire is one of the destructive effects on concrete. During fire, the ingredients of concrete start to dissociate due to their thermal inconsistency [20]. The deterioration of cementitious composites when heated is mainly due to the thermal induced decomposition of the hydrates, and the crack development and propagation [21]. Some of these cracks initiate due to the thermal stresses developed because of thermal gradient, whereas the others initiate due to pore pressure development because of water evaporation [20]. Due to the weak tensile properties of concrete, it cannot sustain these developed cracks leading to degradation in the mechanical strengths and deterioration in the microstructure [22]. Nano reinforcements can restrain the cracks development at nano-level thus is considered one of the promising and effective technique to enhance the high temperature behavior of cementitious composites. Limited research studied the influence of CNTs on the high temperature behavior of cement-based materials [23,24,25,26]. Zhang et al. [23] reported that the effect of CNTs on the residual compressive and flexural strengths of cement paste after heating were most obvious at 400 °C. Beyond this temperature, the CNTs were mostly spalled with the cement matrix at 600 °C. Baloch et al. [24] concluded that incorporating CNTs in concrete enhanced its residual mechanical strengths.

According to the aforementioned literatures, the high temperature behavior of cement-based materials are well established, whereas the influence of CNTs on the thermal characteristics and post-heating behavior of cementitious composites is not fully understood. Thermal properties and post-fire behavior of cementitious composites are essential to predict the ability of concrete structures to survive after exposing to fire. The present research studies the influence of CNTs on phase composition, thermal behavior, microstructure deterioration, and post-heating mechanical strengths of cement mortar.

## 2. Experimental Work

### 2.1. Materials

Ordinary Portland cement, silica sand, tap water, multiwalled carbon nanotubes MWCNTs aquatic solution, and superplasticizer were used in this research to prepare the plain and CNT-reinforced cement mortar. The chemical compositions of the used cement are shown in Table 1. The silica sand was confirmed as meeting ASTM C778-17 Standard and had fines modulus of 2.31, specific gravity of 2.56, and water absorption of 1.87%. The carbon nanotubes aquatic solution was used as received from Nanocyl^®^. Table 2 summarizes the properties of CNTs. Commercially available superplasticizer “PC 485” was used as received from EPSILONE to improve the workability of the mixes.

### 2.2. Mix Proportions, Mortar Casting, and Specimen Preparation

Three batches of mortar were prepared. The first one was plain mortar without CNTs (control). The second batch was prepared with 0.05% of CNTs by weight of cement and was used in compressive tests. The third batch was prepared with 0.2 wt. % of CNTs and was used in flexural tests. These dosages were chosen based on previous study published by the authors [27] concluded that 0.05% and 0.2% are the optimum amount of CNTs to give maximum enhancement in mechanical strengths. For all mixes, 1% superplasticizer by weight of cement was used. Fixed w/c ratio of 0.48 was utilized in all mixes. Detailed mixes design are summarized in Table 3.

ASTM C305 standards were followed to cast the mortar as follows: The cement, the superplasticizer, and the water or the CNTs solution were mixed for 3 min. After that, the sand was added and the ingredients were mixed for three more minutes. Cube molds with 50 mm × 50 mm × 50 mm dimensions and prisms of 40 mm × 40 mm × 160 mm were used to cast the compressive and flexural specimens, respectively. Twenty-four hours later, the specimens were demolded and cured for 28 days in water. Finally, the specimens were removed from water, left at room temperature for 24 h., and then exposed to elevated temperatures.

### 2.3. Heating and Test Procedures

**Figure 1:**
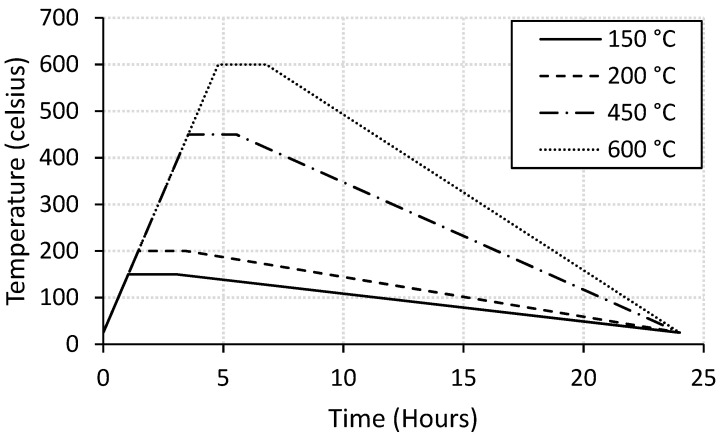
Temperature–time profiles.

Electrical furnace was used to heat the mortar specimens at 25 °C, 150 °C, 200 °C, 450 °C, or 600 °C for two hours according to the heating profile presented in Figure 1. After that, the specimens were left at room temperature to cool down. The control and CNT-reinforced mortar specimens were designated with “Control” and “CNT”, respectively, followed by numbers referring to the exposed temperature. The compressive and flexural strength tests were conducted for three samples of each mix according to the ASTM standards (C109/C109M and C348-18, respectively). After that, the thermal analysis and the microstructural investigation were conducted for selected specimens.

Effect of CNTs on microstructure deterioration of cement mortar after heating was explored through scanning electron microscopy (SEM) analysis. The surface of the extracted fragments were coated by gold before tested using NOVA NanoSEM 450 device according to ASTM C1723-10. X-ray Diffraction (XRD) tests were conducted to qualitatively examine the phase composition and the influence of CNTs on the change of mineralogical contents of the mortars when heated. Small pieces were extracted from the specimens, grinded to powder, and tested using a JSX 3201M (Jeol) spectroscopy device.

Thermal characteristics (heat capacity, heat flow, and phase changes) of plain and CNTs-modified cement mortar were examined by conducting thermogravimetric analysis (TGA) and differential scanning calorimetry (DSC) tests. The fragments extracted from the selected specimens were first grinded to a size of 45 µm then tested using DSC 8500- Perkin Elmer device. Finally, the effect of CNTs on thermal conductivity of cement mortar was examined using TPS 2500s- HotDisk machine.

## 3. Results and Discussion

### 3.1. Phase Composition Analysis Using XRD

**Figure 2:**
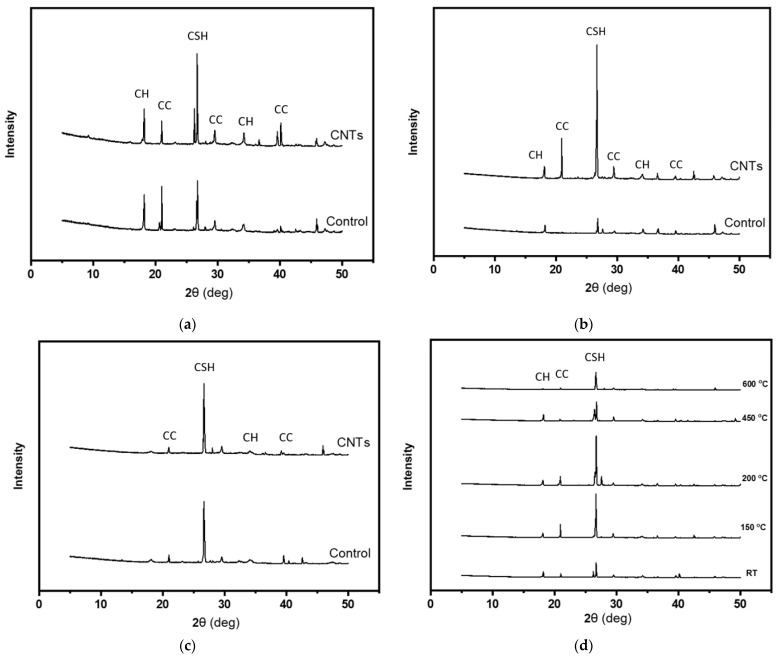
XRD patterns for plain and CNT-reinforced mortar heated at (**a**) RT; (**b**) 200 °C; (**c**) 450 °C; and (**d**) XRD patterns of CNT-reinforced mortar heated at various temperatures.

XRD technique was used to explore the effect of CNTs on the development of cementitious composites’ hydration process, and the deterioration of the hydration products due to heat exposure. The main captured phases in the XRD patterns shown in Figure 2 are calcium hydroxide (CH) at 2θ = 18° and at 2θ = 35°, calcium carbonate (CC) at 2θ = 21° and 2θ = 40°, and calcium silicate hydrate (CSH) at 2θ = 28°. The intensity of the CH and CSH peaks will be used to identify the degree of hydration [23,26]. The XRD patterns of control and CNT-reinforced specimens at room temperature are shown in Figure 2a. The intensity of the CSH peak at 2θ = 28° was increased in the presence of CNTs, indicating positive influence of the CNTs in the hydration process. The enhancement in the hydration process due to the presence of CNTs could be ascribed to the role CNTs play as nucleation spots for the hydration products or to improve the bond between these products [19,25]. For specimens heated at 200 °C, the intensity of the CSH peak at 2θ = 28° of CNT-reinforced cement composites was higher than that of control cement composites as shown in Figure 2b. This finding reflects the role of CNTs in increasing the further hydration process of cement matrix under this elevated temperature. Figure 2c shows similar CSH peaks captured at 2θ = 28° for control and CNT-reinforced mortar heated at 450 °C. This finding indicates that the CNTs have insignificant effect on the hydration process at this heating level. This result is in good agreement with the literature [23]. The influence of the CNTs at this stage may ascribe to their hollow structure which allows to release the high pressure resulted from water evaporation, or from the nano-reinforcing action that plays a major role in bridging the cracks [23]. The effects of high-temperature exposure on the phase composition of the CNT-reinforced cementitious composites are shown in Figure 2d. It is clear that heating the mortar at 200 °C increased the intensity of the CSH peak at 2θ = 28. This finding indicates the further hydration process of the un-hydrated cement particles. The further hydration happened due to the internal autoclaving developed inside the mortar under elevated temperature due to vapor [28]. Further heating the specimens at 450 °C and 600 °C resulted in dehydration process of the CSH gel as can be understood from the reduction in the CSH peak intensity at 2θ = 28. Finally, the CH peak at 2θ = 18 was hardly captured for all heating levels, especially at 600 °C.

### 3.2. Microstructure Deterioration Due to Heating

**Figure 3:**
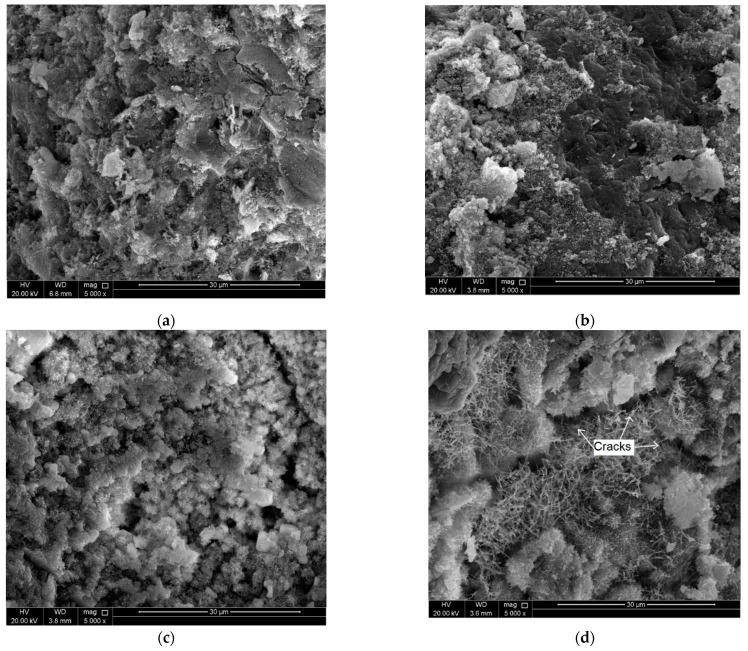
SEM images show the microstructure of mortar with CNTs at (**a**) RT; (**b**) 200 °C; (**c**) 450 °C; (**d**) 600 °C.

**Figure 4:**
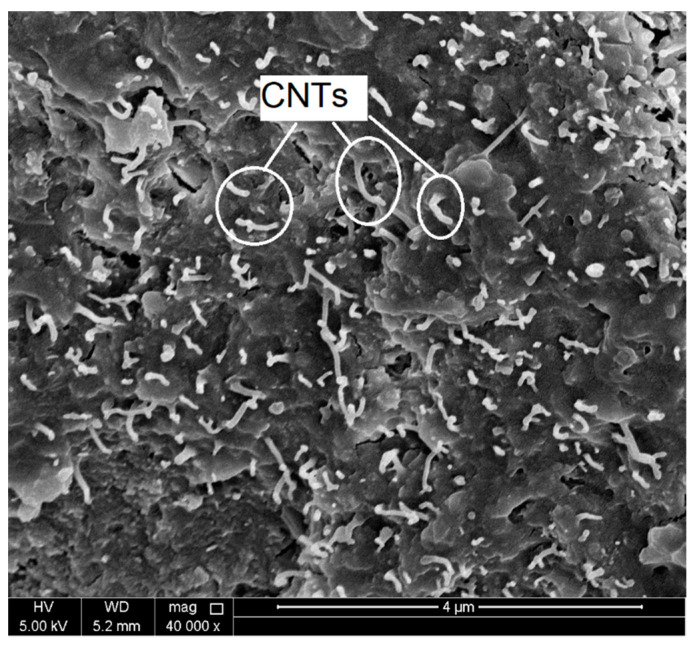
SEM image shows well-dispersed CNTs within the hydration products.

**Figure 5:**
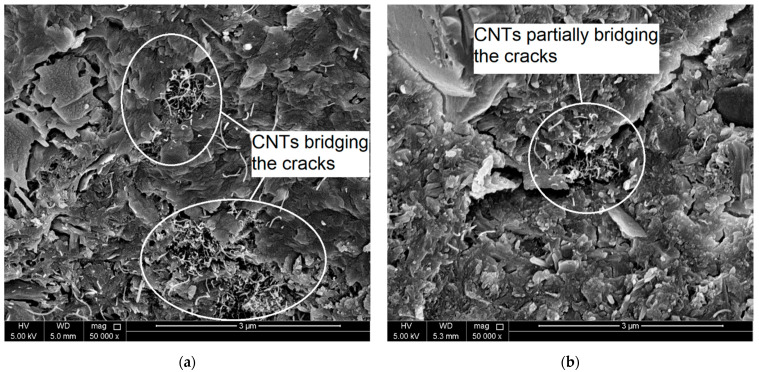
SEM images show the cracks bridging effect of the CNTs for mortar heated at (**a**) 200 °C; (**b**) 450 °C.

In order to investigate the role of CNTs in resisting the deterioration in the cement mortar microstructure when exposed to elevated temperatures, scanning electron microscopy (SEM) analysis was conducted for selected CNT-reinforced mortar specimens before and after heating. For control specimens, CSH, ettringite needles, and CH were captured in the morphology of the microstructure of the mortar, as shown in Figure 3a. Well dispersed CNTs were observed through the hydration products as shown in Figure 4. Insignificant decomposition of the hydration products was noticed due to heating the mortar specimens at 200 °C, as shown in Figure 3b, which shows un-deteriorated microstructure. This finding is consistent with the above-mentioned XRD results and agrees with the literature [23,24]. However, thermal microcracks started to initiate and to propagate due to heating at 200 °C. At this stage, the CNTs filled the voids leading to bridge the cracks and cause a delay of their propagation, as shown in Figure 5a. Further heating of the mortar up to 450 °C resulted in some ill-crystallized or amorphous structures due to the loss of bound water from the decomposition of CSH [23], as shown in Figure 3c. However, no severe dehydration of the CSH was observed where its gel structure did not transfer into crystal structure. In addition, the bridging effect of the CNTs was still obvious, as shown in Figure 5b. Heating the mortar up to 600 °C caused further decomposition of the CSH and CH hydrates and thus caused sever deterioration in the microstructure of the matrix combined with large number of wide cracks as shown in Figure 3d. CNT observation at this heating level was difficult due to the spalling of the CNTs together with hydration products in the matrix [23].

### 3.3. Thermal Behavior Characterization

#### 3.3.1. Thermal Conductivity

**Figure 6:**
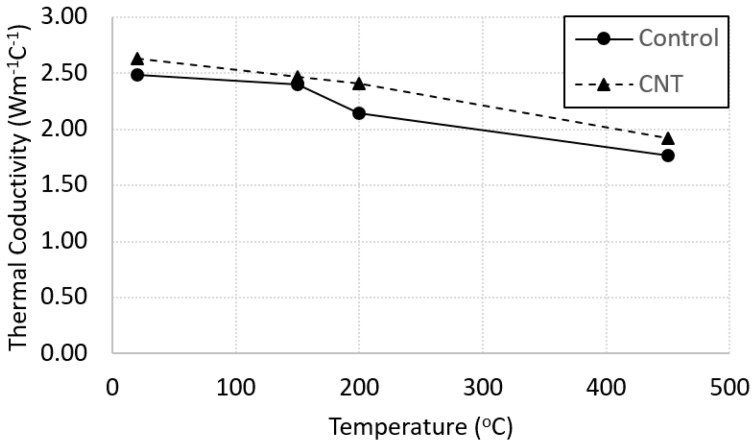
Thermal conductivity of plain and CNT-reinforced specimens as a function of elevated temperatures.

Figure 6 shows the effect of heating on the thermal conductivity of cement mortar with or without CNTs. Heat exposure of cement mortar reduced its thermal conductivity regardless of the presence of CNTs. This finding may be attributed to the deterioration of the microstructure and decomposition the hydration products of the mortar when exposed to elevated temperatures, as proven in the SEM images. However, specimens with CNTs owned higher thermal conductivity than that of control specimens regardless of the heating level. This finding agrees with other results reported in [29] and could be ascribed to: (1) The well distribution of the CNTs, which owned higher conductivity than cement paste [29], through the hydration products; (2) The filling effect of the CNTs, which reduced the voids, thus enhancing the microstructure of the mortar.

#### 3.3.2. Heat Flow Analysis Using DSC

**Figure 7:**
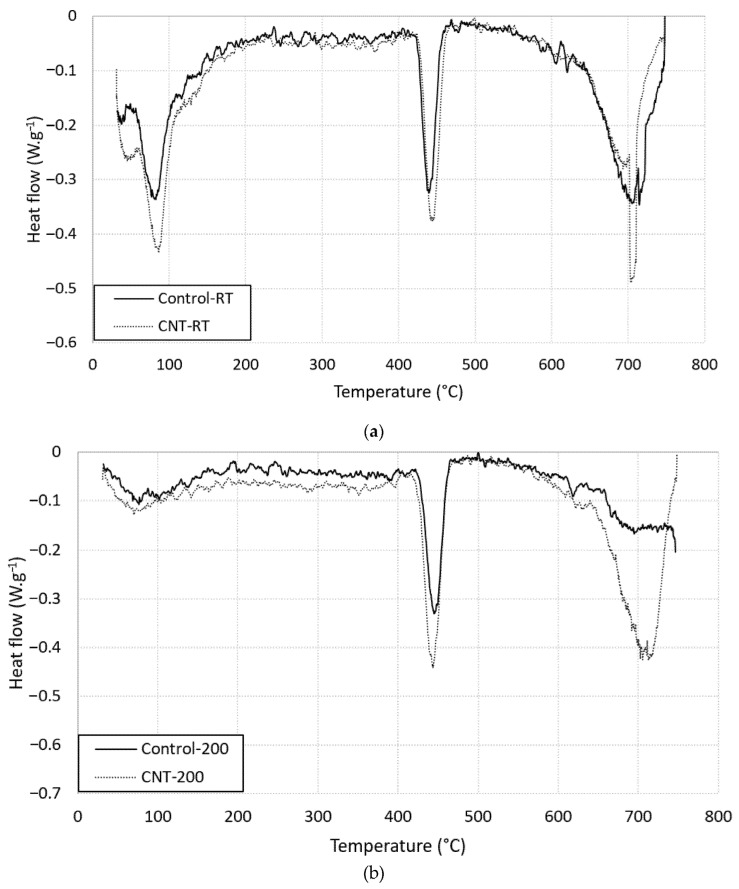
Differential scanning calorimetry (DSC) thermographs for plain and CNT-reinforced mortar (**a**) at room temperatures; (**b**) heated at 200 °C.

Figure 7 shows the DSC thermographs for plain and CNT-reinforced mortar. Three peaks were captured in the graph for unheated mortars at the following intervals: 60–120 °C, 420–475 °C, and 650–750 °C. These peaks refer to the decomposition of CSH, decomposition of portlandite, and decomposition of calcite, respectively [27]. Presence of CNTs caused a shift in the dissociation temperature of the first and second peaks toward higher values. In addition, presence of CNTs increased the area of the first peak which reflected higher heat absorption. These results might be ascribed to the fact that specimens with CNTs contained more free water than plain mortar due to the water absorbed by the CNTs [28], and that these specimens had higher amounts of CSH [29] which supported the XRD results mentioned in the previous section. Heating the specimens at 200 °C did not change the numbers and the location of the observed peaks. However, the presence of CNTs increased the peak value and the area of all observed peaks, as shown in Figure 7bm reflecting more heat absorption needed for products decomposition [28].

#### 3.3.3. Weight Loss Analysis Using TGA

**Figure 8:**
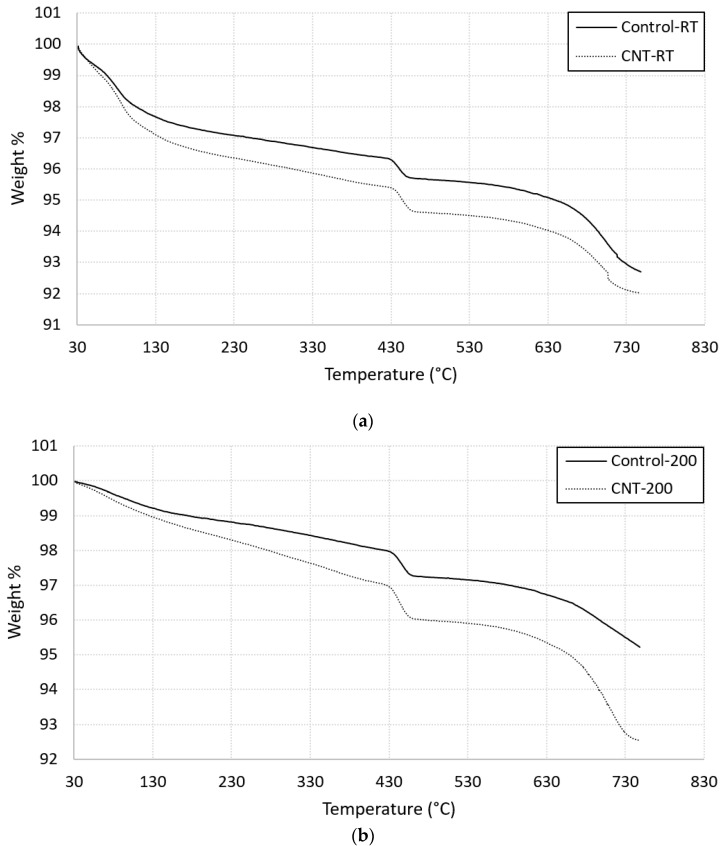
Thermogravimetric profiles for plain and CNTs-reinforced mortar (**a**) at room temperature; (**b**) heated at 200 °C.

Thermographs of cement mortar with and without CNTs are presented in Figure 8. Figure 8a shows three main weight loss steps: 85 °C reflects the decomposition of C-S-H, about 450 °C refers to the Portlandite decomposition, and about 710 °C refers to the decomposition of calcite (CaCO_3_). These results agree with the above-mentioned DSC results. A stiff slope was recognised for specimens with CNTs below 120 °C, indicating more dehydration of pore water [29]. In addition, the presence of CNTs increased the weight loss at 85 °C, which referred to a greater amount of calcium silicates hydrates. The second weight loss step that observed at 450 °C did not affect by the presence of CNTs, but shifted toward higher temperature. This finding is consistence with the above-mentioned DSC results. Figure 8b shows two main weight loss steps for specimens heated at 200 °C. These steps were located at 450 °C, which refers to the decomposition of Portlandite [27], and at 710 °C, which refers to the decomposition of calcite. The presence of CNTs clearly increased the slope of the curves especially below 430 °C. Moreover, an obvious increase in the weight loss step was noticed at this temperature. Finally, the weight loss at 710 °C refers to calcite decomposition and is greatly influenced by the presence of the CNTs.

### 3.4. Mechanical Strengths Degradation

#### 3.4.1. Residual Compressive Strength

Compressive strength results of all test specimens are summarized in Table 4.

**Figure 9:**
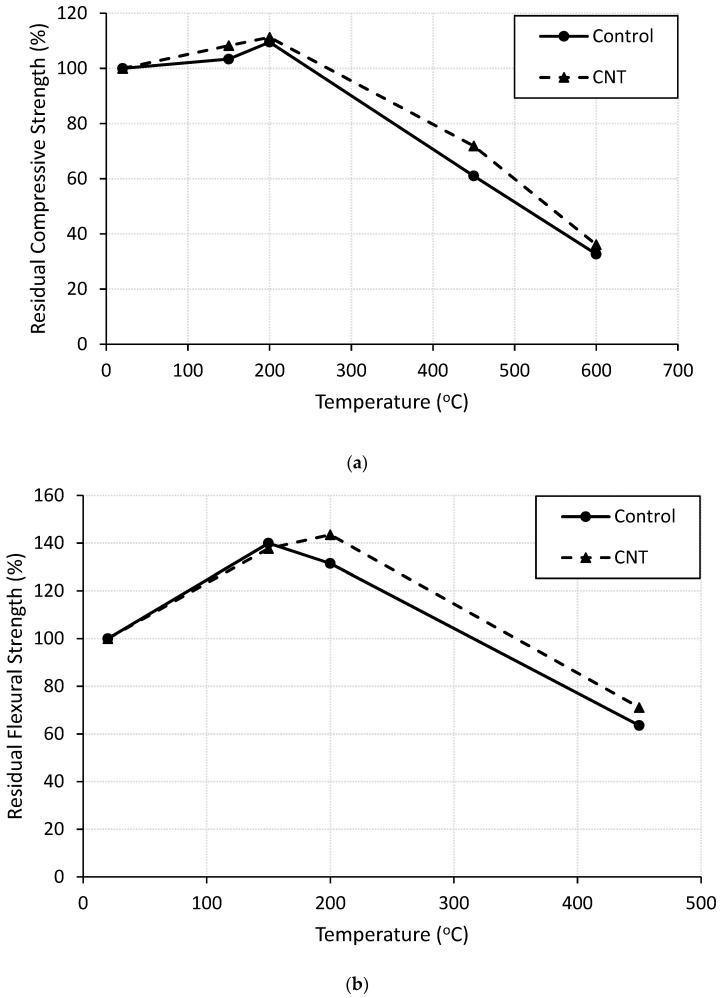
Relative residual strength of control and CNT-reinforced specimens after heating: (**a**) Compressive; (**b**) Flexural.

Compressive strength of unheated control specimen was 30.3 MPa. Adding 0.05 wt. % of CNTs enhanced the compressive strength at ambient temperature by 10%. Similar results were reported in [23,24,25]. The reason for the enhancement may be ascribed to the good dispersion of the CNTs through the hydration products as proven by the SEM images shown in Figure 4. Presence of CNTs enhanced the hydration process by either playing as nucleation spots for the hydration products or improving the bond between these products, which resulted in a denser and more homogeneous microstructure [19,25]. To study the behaviour of CNT-modified cement mortar when exposed to elevated temperatures, the residual strength of heat-damaged mortar was divided by the compressive strength of the corresponding unheated specimens. The results are plotted in Figure 9a as relative residual compressive strength. In general, compressive strength of cement mortar increased with temperatures up to 200 °C. The enhancement in the compressive strength of cement mortar when heated up to 200 °C could be ascribed to the rise in the van der Waals forces between gel particles due to the removal of water content [20,30], or to the continuation of the hydration process as clear in the XRD patterns shown in Figure 2b and mentioned in [23]. Specimens with CNTs showed higher residual strength than control specimens when heated up to 200 °C, as shown in Figure 9a.

The superiority in compressive strength of cement mortar in the presence of CNTs might be due to their ability to bridge the cracks and fill the voids [23]. Further increase in the exposed temperature up to 450 °C caused significant reduction in the compressive strength. This reduction may be attributed to many reasons such as the decomposition of CH and C-S-H as proved by the XRD patterns shown in Figure 2c and mentioned in [24,25], and the growth of the cracks resulting from water evaporation [23,24]. The presence of CNTs significantly improved the residual strength compared to the control specimens. The CNT-reinforced specimen maintains 72% of its original strength when heated at 450 °C compared to 61% for the control specimen. This finding agrees with the literature [23,24,25], and may be credited to the channels’ effect in releasing the steam due to the hollow structure of the CNTs [23]. The process of water molecules passing through inner channels of CNTs was proved by [31,32]. One more reason is the nano-reinforcing action of the CNTs [24] where they play a major role in bridging the cracks, as shown in the SEM graphs in Figure 5. Heating cement mortar at 600 °C severely reduced its compressive strength. Control and CNT-reinforced mortar specimens maintained only 32% and 36% of their original strengths, respectively. This reduction may occur for many reasons such as the severe microcracking in the cement matrix due to heating [25], and the dramatic decomposition of the CSH gel and the calcium hydroxide (CH) [23]. The CSH gel was partly decomposed into C_2_S and C_3_S crystal structures and partly turned into ill-crystallized structure, as shown in the SEM images (Figure 3d). The reduction in the compressive strength of cement mortar due to heating at 600 °C was close for control and CNT-reinforced specimens. This finding could be attributed to the fact that CNTs almost lost their bridging effect [23] due to the severe decomposition of the hydration products, thus, the severe damage of the matrix.

#### 3.4.2. Residual Flexural Strength

Flexural strength results of all test specimens are summarized in Table 4. The relative residual flexural strength was calculated as the ratio between the flexural strengths of heated and unheated specimens, and is plotted in Figure 9b. The flexural strength of control specimen at ambient temperature equals 2.00 kN. Adding 0.2 wt. % of CNTs improved the flexural strength of cement mortar by 13%. The same results were reported in [9,33]. The enhancement might be attributed to the ability of the CNTs to bridge the cracks and to delay their initiation and propagation [10]. Heating control and CNT-reinforced cement mortar specimens up to 200 °C increased their flexural strength compared to the corresponding unheated specimens. The enhancement may be ascribed to the hydration process continuation as is clear in the XRD patterns shown in Figure 2b. Specimens with CNTs showed higher residual strength when heated at 200 °C than control specimens. The main reason beyond this finding could be the ability of the CNTs to bridge the thermal cracks caused by heating and then delaying their propagation during the flexural test as shown in the SEM graphs in Figure 5. In addition, presence of the CNTs with their high thermal conductivity played a major role in distributing the thermal stresses uniformly in the matrix [24,26], therefore, it helped the mortar to resist more stresses and improved its post-heating flexural capacity. Further heating of control and CNT-modified specimens at 450 °C resulted in a significant drop in its flexural strength by 37% and 29%, respectively. This reduction may be ascribed to the decomposition of the hydration products, as shown in the XRD patterns presented in Figure 2c, and the growth of the cracks resulted from internal pressure. Presence of CNTs improved the residual strength due to either their ability to resist the growth of the internal cracks, or their ability to work as channels that allow the steam resulting from water evaporation to pass thorough, thus reducing the pressure.

### 3.5. Visual Inspection and Damage Performance

**Figure 10:**
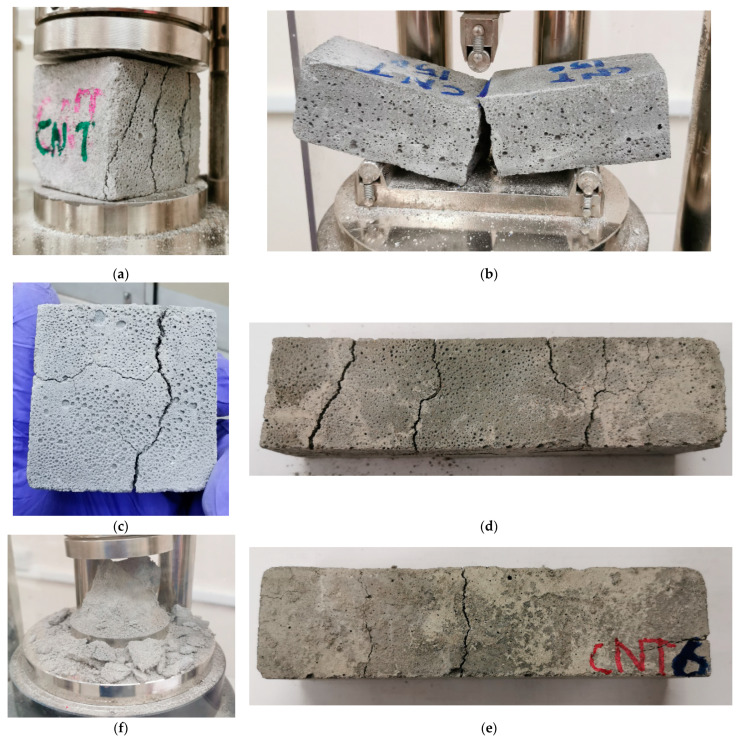
Typical failure mode of specimens heated up to 450 °C: (**a**) compression; (**b**) flexural, thermal cracks caused by heating at 600 °C; (**c**) compression specimens; (**d**) control flexural specimens; (**e**) CNT-reinforced flexural specimens; and (**f**) compression specimens heated at 600 °C after compression test.

During the compression tests, brittle failure was observed for control mortar specimens. Presence of CNTs did not affect the compressive failure mode of the mortar. Typical cracks pattern and failure mode of mortar specimens under compression are shown in Figure 10a. The same failure mode was captured for specimens heated up to 450 °C. In the case of heating level of 600 °C, major wide cracks were visually observed as a result of heating the control and the CNT-reinforced specimens, as shown in Figure 10c. Due to the thermal damage of the specimens, they turned into powder when the mechanical load was applied, as shown in Figure 10f.

The typical failure mode of mortar specimens under flexural loading are shown in Figure 10b. During the test, microcracks were initiated at the interface between the cement particles and the aggregates. The cracks propagated as the load increased until the specimen collapsed with very small deformations. Presence of CNTs delayed the initiation of the cracks but did not affect the failure mode. Same failure mode was observed for specimens heated up to 450 °C. When the temperature reached 600 °C, control mortar specimen owned major and wide cracks due to heating as shown in Figure 10d. The same trend with a smaller number of cracks was observed for the CNT-reinforced specimens, as is clear in Figure 10e. Due to these major cracks, the flexural test could not be carried out, thus the mechanical strength results of specimens heated at 600 °C were omitted.

## 4. Conclusions

Experimental work was conducted in this study to investigate the influence of CNTs on the phase composition, microstructure deterioration, thermal behavior, and mechanical strengths degradation of cementitious composites after exposure to elevated temperatures of 25 °C, 150 °C, 200 °C, 450 °C, and 600 °C for two hours. The following conclusions could be drawn:

1. Presence of CNTs positively affects the hydration process of unheated cement mortar and mortar heated up to 200 °C. Beyond this temperature, the CNTs have no effect on the hydration process.
2. Decomposition of the main hydration products such as CSH and CH is observed when heating the cement mortar at 450 °C, whereas severe deterioration in the microstructure of the mortar is observed at heating level of 600 °C. The CNTs bridge the cracks, delay their propagation, and restrain the deterioration in the microstructure.
3. CNT incorporation enhances the thermal conductivity of the unheated and heat-treated mortar specimens.
4. More heat is needed to decompose the hydration products of cement mortar in the presence of CNTs.

Presence of CNTs significantly enhances the residual compressive and flexural strengths of heated mortar specimens for all studied temperatures.

## Figures and Tables

**Table 1:** Chemical Compositions of Cement.

Compound Name	Content Percentage
CaO	66.4%
SiO_2_	18.4%
Fe_2_O_3_	6.1%
SO_3_	3.0%
Al_2_O_3_	2.2%
MgO	1.4%
Na_2_O	0.8%
LOI	1.5%

**Table 2:** Properties of carbon nanotubes (CNTs).

Property	Value
Average length (µm)	1.5
Average diameter (nm)	9.5
CNTs concentration by weight	3.0%
Carbon purity (%)	90
Surface area (m^2^/g)	250–300

**Table 3:** Mortar Mix Design.

Mortar Batch	Cement	Sand	Water	CNTs	Superplasticizer
	(kg/m^3^)	(kg/m^3^)	(kg/m^3^)	(kg/m^3^)	(kg/m^3^)
1	706.67	1943.33	342.67	-	7.06
2	706.67	1943.33	342.67	0.353	7.06
3	706.67	1943.33	342.67	1.412	7.06

**Table 4:** Mechanical strengths results.

Specimen	Compressive Strength (MPa)	Flexural Strength (kN)
C-RT	30.3	2.00
C-150	31.3	2.80
C-200	33.2	2.63
C-450	18.5	1.27
C-600	9.9	0.21
CNT-RT	33.3	2.25
CNT-150	36.0	3.10
CNT-200	37.0	3.23
CNT-450	23.9	1.60
CNT-600	12.0	NA

## Data Availability

The data presented in this study are available on request from the corresponding author.
